# The *POLG* Gene Polymorphism in Iranian Varicocele-Associated Infertility Patients

**Published:** 2012

**Authors:** Mohammad Mehdi Heidari, Mehri Khatami, Ali Reza Talebi

**Affiliations:** 1 *Department of Biology, Science School, Yazd University, Yazd, Iran*; 2 *Department of Anatomy, Research Clinical Center for Infertility, Shahid Sadughi University of Medical Sciences, Yazd, Iran*

**Keywords:** CAG repeats length, Infertility, Mitochondria, POLG gene, Varicocele

## Abstract

**Objective(s):**

Varicocele is associated with impaired testicular function and male infertility, but the molecular mechanisms by which fertility is affected have not been satisfactorily explained. The aim of our study was to investigate whether or not the *polymerase gamma* (*POLG*) polymorphism is associated with Iranian varicocele patients.

**Materials and Methods:**

We determined the *POLG* CAG repeat length in DNA samples extracted from 40 varicocele patients and 30 control subjects by PCR-denature polyacrylamide gel electrophoresis and sequencing.

**Results:**

The distribution of the CAG repeat length in varicocele patients showed no notable difference from that in control subjects, but we found a significant statistical inverse correlation between 10/10 and 10/#10 genotypes and varicocele grade.

**Conclusion:**

These findings indicate that *POLG* CAG repeats may affects the varicocele grade, but the mechanisms remain to be elucidated.

## Introduction

The varicocele is the most common cause of male infertility worldwide. Varicoceles have been found in 15% of the normal male population and in up to 40% of patients with male infertility (1, 2). In approximately 70% of patients with secondary infertility, a varicocele is an underlying cause (3). The theory behind the reason why varicocele results in impaired spermatogenesis includes oxygen deprivation, poor venous drainage that leads to impaired drainage of gonadotoxins from the testis, and increased oxidants within the semen (4). Allamaneni and colleagues reported a positive correlation between seminal reactive oxygen species (ROS) or ‘‘oxidant’’ levels and varicocele grade (5). They demonstrated that higher seminal ROS levels are seen in men with grade II and III varicoceles compared with men with grade I varicoceles. The mitochondrial oxidative stress is thought to be involved in the pathogenesis of this disease (5, 6). Talebi *et al* showed that the varicocele samples contain a higher proportion of spermatozoa with abnormal DNA and immature chromatin than those from fertile men (7). Understanding the pathophysiology, treatments, and outcomes of a varicocele and varicocele repair has evolved significantly over the several past decades.

The nuclear catalytic subunit of *polymerase gamma* (*POLG)* is the only polymerase in humans that is known to be located in mitochondria and has been proposed as the replicase of mitochondrial DNA (8). In its coding region lies a short polyglutamine tract, which is absent in *POLG *of mouse or Drosophila (9, 10). Microsatellite repeats have been shown to evolve in length during the course of primate evolution and show considerable variation between individuals. Alterations in the CAG repeat region have been found to be associated with idiopathic sporadic Parkinson (11) and Friedreich ataxia (12). 

Many repeat length alleles different from 10 (ranging from 6–15) have been found and are considered mutants (13). Ten copies of CAG repeat were found to be uniformly high (0.88) in different ethnic groups (14), hence are considered as the common allele whereas the mutant alleles (other than 10 ⁄ 10 CAG repeats) were found to be associated with oligospermia male infertility (15). The authors hypothesized that the presence of mutated alleles would lead to a suboptimal mtDNA polymerase resulting in the accumulation of mutations in the mtDNA that would cause impaired energy metabolism of the spermatogenic cells and finally bring about a disturbance of sperm production and/or differentiation. The aim of the present study was to assess whether the *POLG* polymorphism are associated with Iranian varicocele-associated infertility patients.

## Materials and Methods

***Patients***

The study group included forty Iranian infertile male patients with varicocele. The varicocele diagnosis was made, by the same urologist, for the patients in standing position and via scrotal palpation in a temperature controlled room (23 °C). Semen analysis was performed according to WHO criteria (16). Thirty healthy donors with proven fertility who had a successful pregnancy within the last 12 months and normal spermogram at the time of study were selected as control group. All of the patients and control group were informed of the aims of the study and gave their informed consents to the genetic analysis. The institutional review board at Yazd University of Medical Sciences approved this prospective study.

***The amplification of the POLG gene and Sequencing***

DNA was isolated from peripheral blood samples using a DNA extraction kit (Sinaclone, Tehran, Iran). The CAG-repeat region of the *POLG* gene was amplified by PCR with two primers (5'-GGTCCCTGCACCAACCATGA-3' and 5'-CTTGCCCGAAGATTTGCTCGT-3') that was published previously (17). PCR of each sample was set in a 0.5 ml tube using 100 mg of total DNA, 10 pmol of each primer, 200 mmol of dNTPs, 1X PCR buffer containing 2.5 mmol MgCl_2_ and 1 U Taq DNA polymerase (Roche Diagnostics, Mannheim, Germany). Cycling condition used for the amplification was as follows: initial denaturation at 94 °C for 5 min followed by 30 cycles of denaturation for 1 min at 94 °C, annealing for 1 min at 60 °C and extension for 1.5 min at 72 °C (final extension for 10 min).

**Figure 1:**
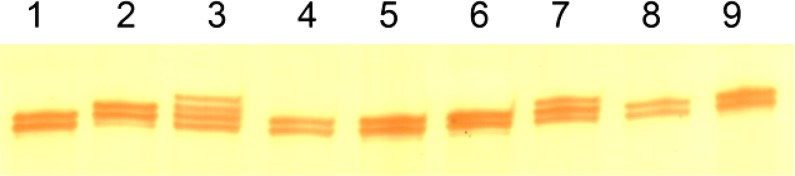
Genotyping of *POLG* CAG repeat by 8% denature polyacrylamide gel in patients. Lane 1, 4-6, 8 and 9: 10/10; lane 2 and 7: 10/11; lane 3: 10/12.

PCR products were resolved on 1-mm thick, 8% polyacrylamide gels by electrophoresis under denaturing conditions. Gels were stained with silver to reveal the *POLG *CAG repeat length (Figure 1). Any DNA fragments showing differences in banding patterns between the control and patient were sequenced to identify the exact mutations. The nucleotide sequence of the amplicon was directly determined by automated sequencing 3700 ABI machine (Macrogene Seoul, South Korea).

***Statistical analysis***

The t-test was applied to determine whether the frequencies of different genotypes in varicocele patients and control groups were significantly different. Values of *P*< 0.05 were regarded as statistically significant. Data were expressed in mean±SD. The statistical analysis was performed using the GraphPad Prism software (GraphPad Software, Inc. USA). 

## Results

A total number of 70 individuals (40 varicocele patients and 30 fertile controls) were examined. Forty patients had: (a) Grade I (n=15); (b) Grade II (n=13); (c) Grade III (n=12). The mean age of infertile men with varicocele and normal controls was (Mean±SD) 31.58±6.16, 31.26±5.31 respectively (*P=*0.8204). 

Sperm parameters including sperm count, rapid, slow, nonprogressive motility and immotile and sperm morphology in three groups are listed in Table 1. Sperm count, rapid motility and the rate of normal sperm morphology was significantly lower in the varicocele groups than in the fertile group (*P* =0.0003, *P* =0.0001, *P* =0.0001 respectively), but slow, nonprogressive and immotile motility between varicocele and fertile group was notable different (*P= *0.1972, *P= *0.1151, The frequency of the *POLG* gene CAG repeats in the two study populations are given in Table 2. The predominant allele in control subjects had 10 consecutive CAG repeats. This allele was found at similar frequency in control subjects (77.5%) and varicocele patients (83.8%) (*P*= 0.6425). The observed homozygosity values for the prevalent allele are close to equilibrium predictions. Other alleles of 9, 11 and 12 trinucleotide repeats were detected, but no larger, expanded repeats were found in the control subjects and varicocele patients.

**Figure 2:**
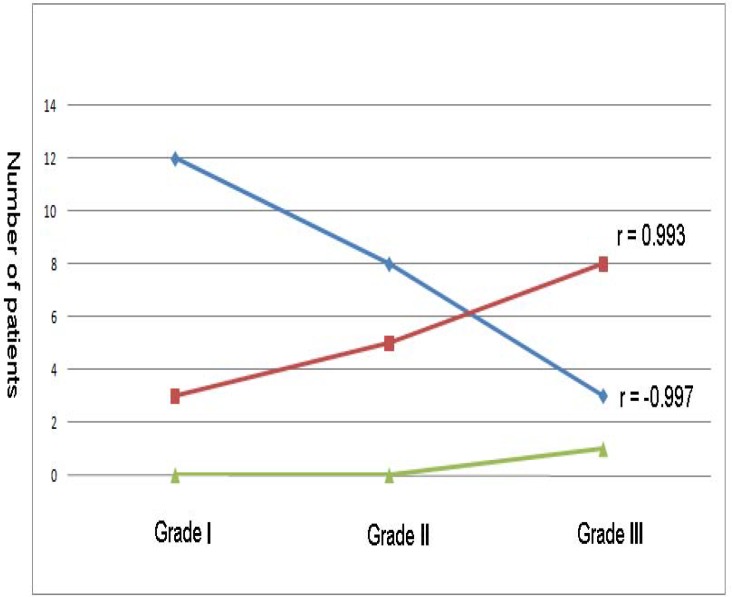
The Correlation between *POLG* genotype and Varicocele grade**.**

## Discussion

Depletion and rearrangements of mtDNA, including those caused by mutations in other regions of the *POLG* gene, are often manifested as serious diseases in humans, e.g. progressive external ophtalmoplegia (18). The molecular mechanism of this impairment remains to be elucidated.

Varicocele is the most common reversible cause of infertility. While the exact pathophysiologic mechanism underlying the Varicocele is the most common reversible cause of infertility. While the exact pathophysiologic mechanism underlying the hazardous effects of a varicocele on spermatogenesis and male fertility is not completely understood yet; however, most scientific evidence supports that both venous reflux and testicular temperature elevation seem to play an important role in the development of varicocele induced testicular dysfunction (19, 20).

**Table 1:** Distribution of semen quality in patients.

		Varicocele			Control (n=30)	*P*-value^a^
Variables	Grade I (n=15)	Grade II (n=13)	Grade III (n=12)	Overall (n=40)		
Age	34.50±5.82	30.55±5.03	26.63±5.99	31.58±6.16	31.26±5.31	0.8204
Count (mil.ml^-1^)	62.17±45.77	57.00±31.76	53.21±27.23	57.46±38.57	98.76±53.03	0.0003
Rapid motility (%)	17.51±9.41	13.48±7.94	12.78±5.21	14.59±7.57	23.24±10.05	0.0001
Slow motility (%)	36.17±9.90	40.64±11.78	32.53±10.20	36.44±10.93	33.45±7.16	0.1972
Nonprogressive motility (%)	11.41±5.40	12.92±3.11	13.10±3.67	12.47±3.99	10.98±3.69	0.1151
Immotile motility (%)	34.91±17.75	32.8±18.86	41.59±10.34	36.43±15.84	32.43±9.35	0.2228
Normal morphology (%)	28.08±12.98	18.09±12.07	23.68±7.35	23.63±11.66	44.52±14.46	0.0001

a: Difference between varicocele (n=40) and control (n=30) group

Numerous studies have indicated an association between different polymorphisms, mutations or deletions in the mitochondrial genome and sperm dysfunction (21). To explore the influence of genetic factors, which associate with varicocele, we investigated the possible role of CAG repeats in *POLG* gene in these patients. Mutation in the *POLG* gene might introduce mutation in the mtDNA during replication, which eventually could affect the motility of the spermatozoa (-). *POLG *gene comprised variable number of CAG repeats, of which 10 copies of CAG repeats were considered as a common allele (14).

The distribution of the CAG repeat length in varicocele patients showed no notable difference from that in control subjects (Table 2). Krausz *et al*, Aknin-Seifer *et al* and Rani *et al* also fail to find any association between CAG repeat variation and male infertility among Italian, French and Indian populations, respectively (13, 24, 25). 

**Table 2:** *POLG* genotype of varicocele patients and controls

Allele (number of CAG repeats)	Num. of alleles in varicocele patients					Number of alleles in controls	*P-* value^a^
	Grade I		Grade II	Grade III	Total		
8	0		0	0	0 (0%)	0 (0%)	-
9	1		3	1	5 (6.25%)	3 (4.8%)	0.7322
10	29		21	14	62 (77.5%)	54 (83.8%)	0.6425
11	2		2	7	11 (13.75%)	4 (13%)	0.2047
12	0		0	2	2 (2.5%)	0 (0%)	0.2156
Genotype	Number of varicocele patients					Number of controls	
	Grade I	Grade II		Grade III	Total		
10/10 Homozygous	12	8		3	23 (57.5%)	21 (70%)	0.6107
10 /≠10 Heterozygous	3	5		8	16 (40%)	9 (30%)	0.5497
≠10/≠10 Homozygous + Heterozygous	0	0		1	1 (2.5%)	0 (0%)	0.3890
Num. of samples	15	13		12	40	30	

a: Difference between varicocele and control group

Although our results show that somatic instability of the *POLG *CAG repeat is not a main factor in pathogenesis of varicocele, but we found a statistically significant correlation between 10/10 genotype with varicocele grade (r= 0.993) and a statistically significant inverse correlation between 10/#10 genotype with varicocele grade (r= -0.997) (Figure 2). These findings suggested that the frequency of 10/#10 genotype increased in grade III and the frequency of 10/10 genotype increased in grade I. 

## Conclusion

Thus, although we did not find any association between the *POLG* gene polymorphism and Iranian varicocele patients, but we suggest that *POLG* CAG repeat extensions is a contributing genetic risk factor that affects varicocele grade. The possible mechanisms remain elusive and require further studies. 
